# A case report of acute Leriche syndrome: aortoiliac occlusive disease due to embolization from left ventricular thrombus caused by myocarditis

**DOI:** 10.1186/s12872-021-02031-4

**Published:** 2021-04-30

**Authors:** Abudi Mohamed, Gustav Mattsson, Peter Magnusson

**Affiliations:** 1 Centre for Research and Development, Uppsala University/Region Gävleborg, 801 87 Gävle, Sweden; 2 Cardiology Research Unit, Department of Medicine, Karolinska Institutet, 171 76 Stockholm, Sweden

**Keywords:** Aortoiliac occlusive disease, Cardiac embolization, Case report, Embolectomy, Heart failure, Leriche syndrome, Myocarditis

## Abstract

**Background:**

Leriche syndrome is the triad of symptoms consisting of claudication, erectile dysfunction, and absence of femoral pulses. Inflammatory disease of the heart muscle, myocarditis, may occur because of immune system activation, drug exposure or infections.

**Case presentation:**

A 31-year-old man with no previous medical history presented to the emergency department with acute back pain that had started suddenly during weightlifting, which was initially misdiagnosed as spinal disc herniation. The patient returned four hours later and a thoracoabdominal computed tomography showed a large thrombus in the aortoiliac region creating a total occlusion. Vascular surgery with embolectomy was immediately performed. Further investigation with echocardiography revealed deteriorated systolic dysfunction with marked hypokinesia and two large thrombi in the left ventricle. Cardiac magnetic resonance imaging showed late contrast enhancement of the inferolateral and septal regions, which indicated a recent myocarditis.

**Conclusion:**

Myocarditis can result in multiple embolization with diverse organ manifestation including total occlusion of the aortoiliac arteries, which required urgent embolectomy.

**Supplementary Information:**

The online version contains supplementary material available at 10.1186/s12872-021-02031-4.

## Background

Aortoiliac occlusive disease was first described by Robert Graham in 1914, but it was not until some years later that Henri Leriche described the triad of symptoms as a syndrome and thus it was given his name. Leriche syndrome is the triad of claudication, impotence, and decreased/absence of femoral pulses due to aortoiliac occlusion [1]. Risk factors include hyperlipidaemia, hypertension, male sex, diabetes mellitus, and smoking. In most cases this is caused by chronic development of atherosclerotic plaques which in turn might cause thrombus formation in the aorta creating an occlusion [2]. Historically, open repair have been the primary choice for aortoiliac disease (occlusion and stenosis) with excellent patency rates at 72–90% at 10 years. However, with increasing endovascular skills and equipment this has been challenged [3]. The vascular society however still recommends open repair in the case of aortoiliac occlusion [4].

While myocarditis most often has a benign course, it is also considered to be the cause of 4% of heart failure in the young and 0.5% in the elderly [5].

Because myocarditis often presents with unspecific symptoms such as chest pain, dyspnoea, fever, and palpitations it may go unnoticed and the incidence in the general population is therefore difficult to estimate [6, 7]. Younger men seem to be more susceptible to myocarditis than women [8].

There are several causes of myocarditis including viral agents, toxic substances, and immune mediated responses [6]. Magnetic resonance imaging of the heart is widely used for non-invasive assessment of myocardial inflammation in patients with suspected myocarditis [9].

## Case presentation

**Fig. 1:**
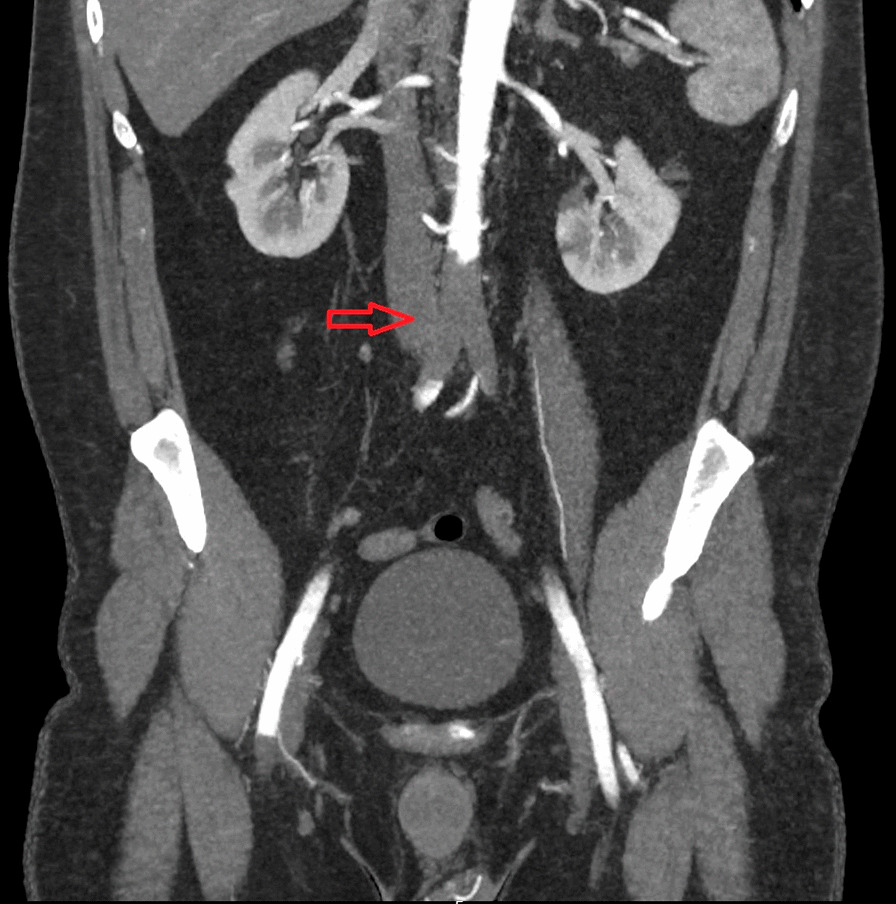
Thoracoabdominal computed tomography in coronal view during visit in the emergency department showing a massive thrombus in the aortoiliac region

A 31-year-old man without previous medical history presented to the emergency department with acute back pain that had started suddenly during weightlifting. Initially, spinal disc herniation was suspected by the orthopedic surgeon and the patient was discharged. He returned four hours later with pain and a sense of numbness in the right leg and an absence of femoral pulses bilaterally. A thoracoabdominal computed tomography (CT) angiography (Fig. 1) showed embolism to the left renal artery, splenic artery, and a massive thrombus in the aortoiliac arteries. Then the CT scan was elongated to include the lower limbs, which revealed embolism in the right superficial femoral artery. The vascular surgeon decided to perform an acute embolectomy.

Laboratory tests for C-reactive protein, hemoglobin, and thrombocytes were normal, but leukocytes were slightly elevated at 13.3 × 10^9^/L. The coagulation samples showed PK INR level of 1.1, APTT 32 s, fibrinogen 2.5 g/L, D-dimer 1.55 mg/L, and antithrombin 1.08 kIU/L. Because this occurred during the coronavirus disease 2019 (COVID-19) pandemic, polymerase chain reaction for SARS-CoV-2 was performed and was negative.

The surgical procedure confirmed embolism of both acute and chronic appearance; soft black thrombi suggesting acute formation and white hard cylindrical (shape of the iliaca vessels) suggesting chronic forms. During surgery, a medial and lateral lower leg fasciotomy was also performed on the right side in order to prevent post-operative compartment syndrome.

**Fig. 2:**
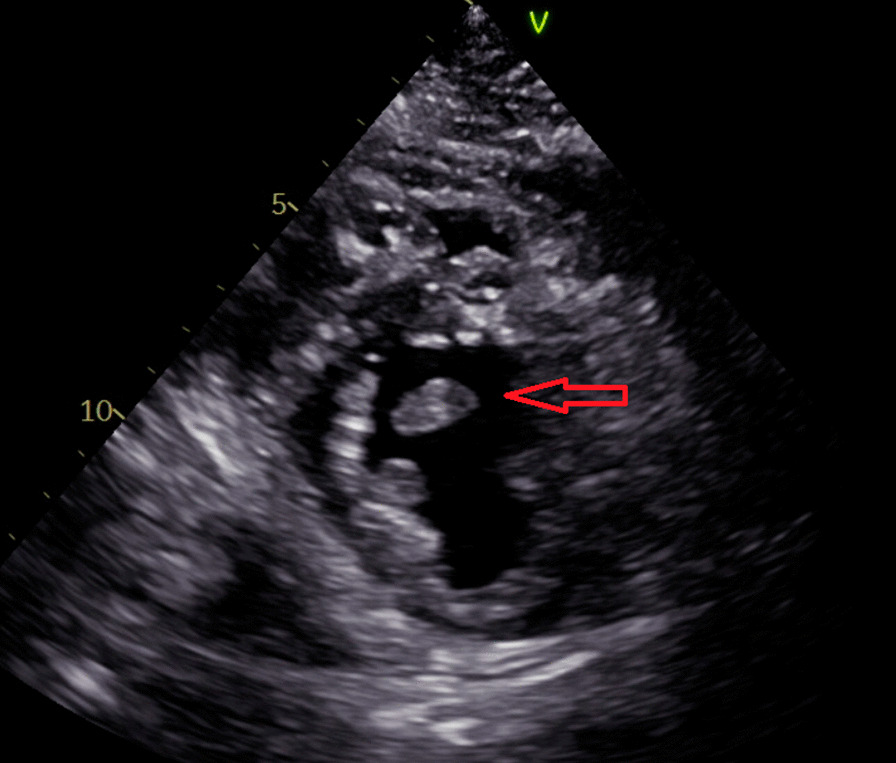
Echocardiography in parasternal short axis view performed post-operatively showing a large thrombus in the left ventricle

**Fig. 3:**
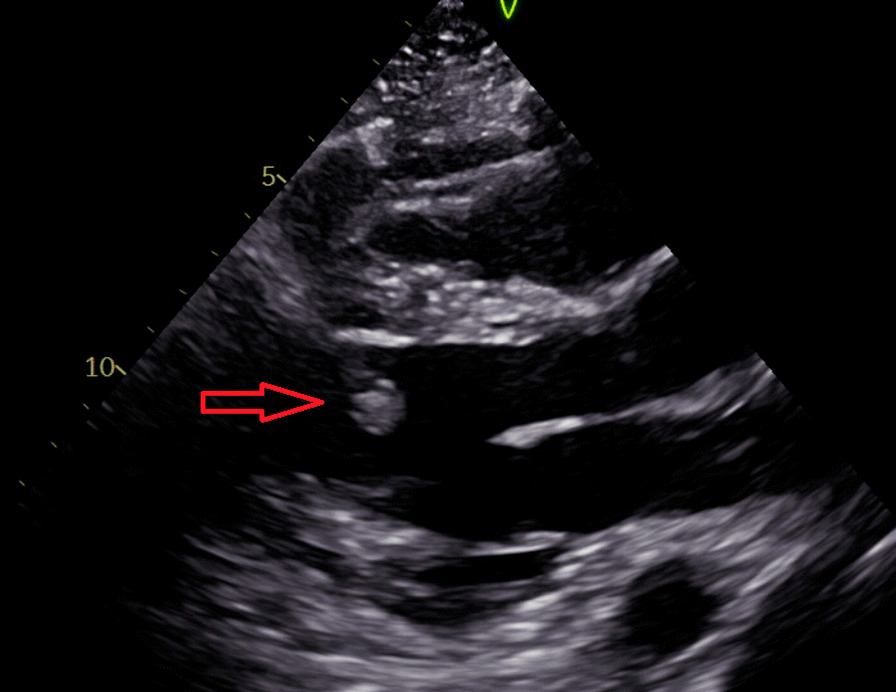
Echocardiography in parasternal long axis view performed post-operatively showing a large thrombus in the left ventricle

Following surgery an echocardiography (Additional files 1–4: Videos S1–S4) demonstrated left ventricular inferior hypokinesia, an ejection fraction of 30–40%, and two large thrombi in the left ventricle (Figs. 2 and 3). The ECG showed sinus rhythm with minor intraventricular conduction delay but was otherwise unremarkable. During a four day stay at the intensive care unit the patient was initially treated with low molecular weight heparin with a dose of 5000 IU twice daily (because of recent surgery) and this was slowly increased to full dose. The hormone T4 was 15.5 pmol/L, T3 2.6 pmol/L, thyroid stimulating-hormone 0.65 mIU/L, and testosterone 6.1 nmol/L. Troponin T was elevated at 75 ng/L, N-terminal pro-B-type natriuretic peptide was 310 ng/L, and anti-SARS-CoV-2(ECLIA) was negative.

**Fig. 4:**
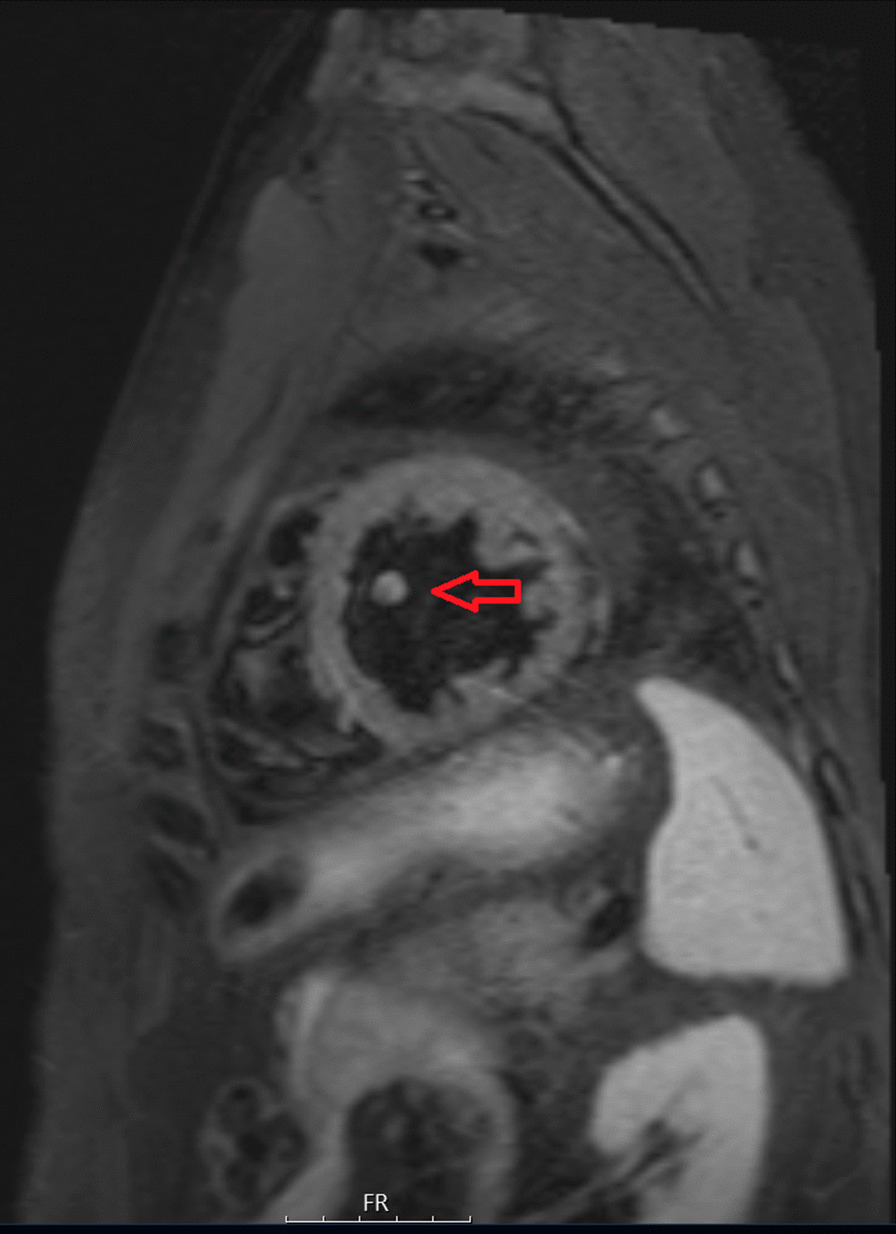
Cardiac magnetic resonance image in short axis view performed on the second day of admission demonstrating a thrombus in the left ventricle

**Fig. 5:**
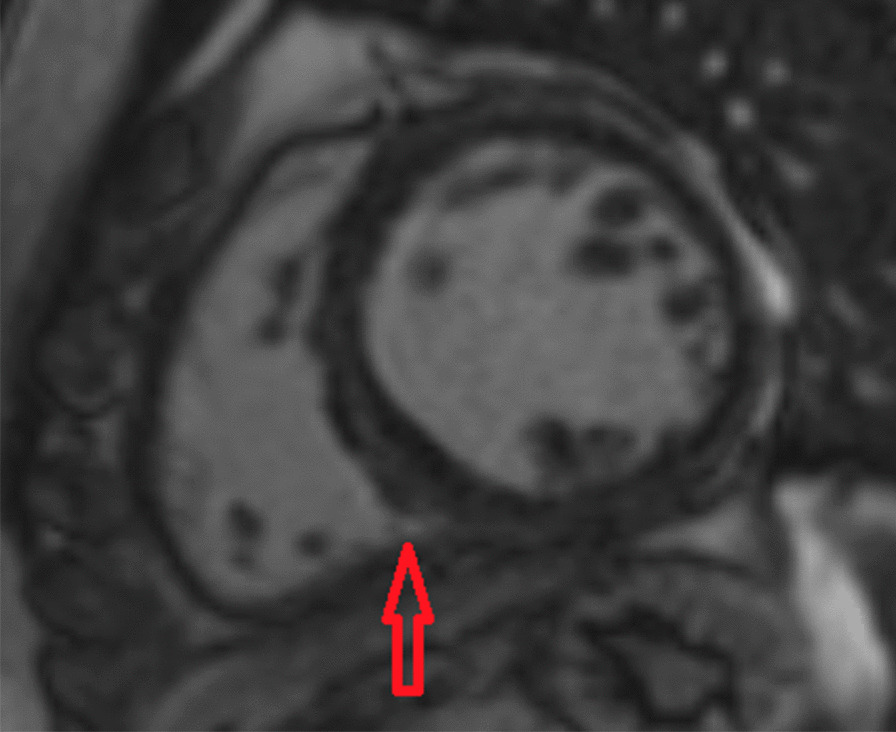
Cardiac magnetic resonance image in short axis view showing subepicardial late gadolinium enhancement in the interventricular septum suggesting previous myocarditis

**Fig. 6:**
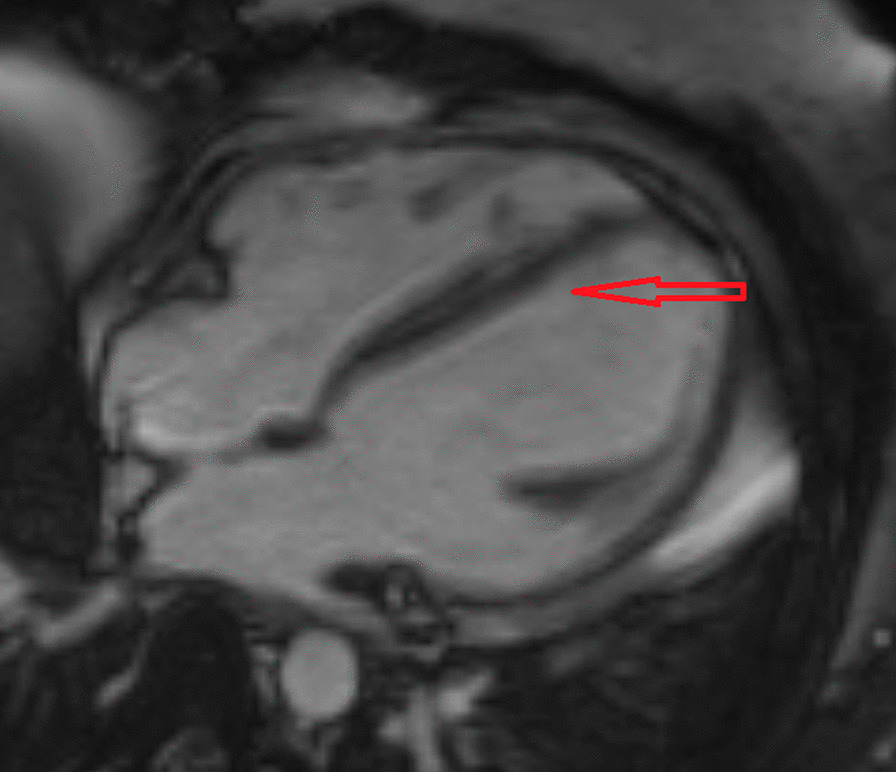
Cardiac magnetic resonance image in four chamber view showing subepicardial late gadolinium enhancement in the interventricular septum suggesting previous myocarditis

At the second day after admission a cardiac magnetic resonance imaging assessed reduced ejection fraction (44%), it also showed a thrombus in the left ventricle (Fig. 4) and late gadolinium enhancement of the inferolateral and septal regions (Figs. 5 and 6), which indicated recent myocarditis. Two large thrombi, previously seen on echocardiogram, were visualized in the midventricular-apical region. A more detailed patient history was taken, and the patient reported an episode of fever, chest pain, and shortness of breath two months earlier but never sought medical attention for this. This was interpreted as myocarditis likely due to viral infection.

Successful suture of the fasciotomy was performed six days after the primary surgery. The patient was transferred to an internal medicine ward. Tests for coagulopathies were taken and included haptoglobin which had a level of 1.45 g/L and homocysteine with a level of 28 µmol/L (slightly elevated). Moreover, S-anti-GBM, S-anti-myeloperoxidas, and S-anti-proteinase 3 were normal.

The patient was negative for mutations in JAK2, CALR, MPL, factor II, and V test for paroxysmal nocturnal hemoglobinuria was negative and so were the tests for protein C, protein S, antiphospholipid antibodies (anticardiolipin, anti-beta2-glycoprotein I, and lupus anticoagulant).

Magnetic resonance imaging of the brain demonstrated a non-acute infarct in the left parietal lobe. Ultrasound of the carotid and vertebral arteries were normal. A second echocardiography, performed 11 days after the first, showed slight improvement of left ventricular systolic function, but two thrombi were still present. Apixaban 5 mg twice daily was initiated, but subsequently replaced with warfarin because the thrombi remained at follow-up. ECG showed premature ventricular complex. Bisoprolol and candesartan were titrated, prescribed and the patient scheduled for further follow-up in the cardiology department. Six months after hospital discharge an echocardiogram showed improvement of left ventricular function with an ejection fraction of about 45% and resolution of the thrombi.

## Discussion and conclusions

The severity and prognosis of myocarditis varies greatly, from asymptomatic episodes with spontaneous improvement to severe heart failure or other cardiac complications. In the acute phase systolic dysfunction, ventricular arrhythmias and sudden deaths may ensue and in the chronic phase it can result in heart failure, which may require advanced treatment such as left ventricular assist devices and transplantation in severe cases [10].

Management of myocarditis includes treatment of the underlying etiology, alleviation of symptoms, and if needed management of arrhythmia and heart failure according to general guidelines [11, 12]. The most common cause in developed countries is viral infection [13]. Generally there is no specific treatment for myocarditis, however in cases of certain infectious agents, antiviral or antibiotic treatment can be an option [6]. Optimal management of left ventricular thrombosis remains uncertain. It is a major risk factor for systemic thromboembolism. Although anticoagulants should be used, optimal pharmacological treatment for patients with chronic left ventricular thrombosis is unknown [14]. Limited data exist on the off-label use of non-vitamin K oral anticoagulants in this situation [11]. Left ventricular thrombosis is usually caused by myocardial infarction and non-ischemic cardiomyopathies but has been reported in myocarditis [11, 15].

An acute Leriche syndrome is an extremely rare condition with high risk of morbidity and mortality. Diagnoses is usually made by CT, or in non-acute cases magnetic resonance imaging. While Leriche syndrome is typically caused by chronic atherosclerotic plaque with thrombus formation, cardiac embolization has been described, for example paradoxical embolization due to patent foramen ovale [16].

In the case report presented, there was difficulty in arriving at the correct diagnosis due to lack of typical symptoms and the complexity of the underlying causative pathway. The patient presented with acute back pain but with no other symptoms at the first visit in the emergency department resulting in the incorrect diagnosis of spinal disc herniation. It was not until a couple of hours later when the patient returned complaining of pain and sensory loss in the right leg that that vascular pathology was suspected due to absence of femoral pulses. This illustrates that it is sometimes difficult to separate vascular ailments from neurological [17]. In fact, in 1948 Leriche et al. stated that “One should never diagnose ‘neuritis’ or a ‘polyneuritis’ of the lower limbs, unless one has carefully examined the femoral pulses and the oscillometric curve” [1]. It was not until after an echocardiogram had been performed, which showed hypokinesia and left ventricular thrombosis, that there was any suspicion of myocarditis. The patient had no symptoms at presentation that gave a clue to the existence of cardiac disease. However, retrospectively it is likely that the episode of fever, chest pain, and shortness of breath two months earlier were symptoms of acute myocarditis and the intraventricular conduction delay seen on ECG at admission was caused by this episode.

Diagnostic work-up found no coagulopathy. Due to the myocarditis not being deemed active there was no indication for endomyocardial biopsy and thus no there was no diagnosis of the specific etiology. There were no signs of systemic disease such as vasculitis and therefore the cause was likely viral infection. No atherosclerotic plaques could be seen in the aorta or other arteries, neither with computed tomography nor during surgery. The thrombosis in the aortoilliac region was thus deemed to be due to cardiac embolization, this was strengthened by multiple other systemic emboli. It has been described that COVID-19 can result in a hypercoagulable state which might result in thrombus formation [18]. However, tests both for the COVID-19 virus and antibodies were negative.

In conclusion, an episode of myocarditis resulted in left ventricular thrombosis that later gave systemic embolization including embolus to the renal artery, splenic artery, cerebral artery, and to the distal aorta creating an acute Leriche syndrome causing a life-threatening ailment needing acute vascular surgery.

In the rare event of acute Leriche syndrome, it is of uttermost importance that a thorough history is taken in addition to careful vascular and neurological examination. It should also be highlighted the importance of a diagnostic workup, including echocardiography, with regards to cardiac embolization as the cause of systemic embolization, especially in the case of multiple emboli.

## Supplementary Information

**Additional file 1.** First echocardiography exam: parasternal short axis view showing thrombus.

**Additional file 2.** First echocardiography exam: apical four chamber view showing midventricular and apical thrombi.

**Additional file 3.** First echocardiography exam: apical four chamber view showing left ventricular thrombus.

**Additional file 4.** First echocardiography exam: apical two-chamber view showing midventricular thrombus.

## Data Availability

All relevant data supporting the conclusions of this article is included within the article.

## Abbreviations

CT: Computed tomography
COVID-19: Coronavirus disease 2019
